# Atmospheric Water Harvesting: Role of Surface Wettability and Edge Effect

**DOI:** 10.1002/gch2.201700019

**Published:** 2017-06-23

**Authors:** Yong Jin, Lianbin Zhang, Peng Wang

**Affiliations:** ^1^ Water Desalination and Reuse Center Division of Biological and Environmental Science and Engineering King Abdullah University of Science and Technology Thuwal 23955‐6900 Saudi Arabia; ^2^ Key Laboratory of Materials Chemistry for Energy Conversion and Storage (HUST) of Ministry of Education School of Chemistry and Chemical Engineering Huazhong University of Science and Technology Wuhan 430074 China

**Keywords:** condensation, contact angle hysteresis, edge effect, water collection, wettability

## Abstract

Atmospheric water is emerging as an important potable water source. The present work experimentally and theoretically investigates water condensation and collection on flat surfaces with contrasting contact angles and contact angle hysteresis (CAH) to elucidate their roles on water mass collection efficiency. The experimental results indicate that a hydrophilic surface promotes nucleation and individual droplets growth, and a surface with a low CAH tends to let a smaller droplet to slide down, but the overall water mass collection efficiency is independent of both surface contact angle and CAH. The experimental results agree well with our theoretical calculations. During water condensation, a balance has to be struck between single droplet growth and droplet density on a surface so as to maintain a constant water droplet surface coverage ratio, which renders the role of both surface wettability and hysteresis insignificant to the ultimate water mass collection. Moreover, water droplets on the edges of a surface grow much faster than those on the non‐edge areas and thus dominate the contribution to the water mass collection by the entire surface, directly pointing out the very important role of edge effect on water condensation and collection.

## 1 Introduction

Atmospheric moisture is abundantly present in our ambient air and is emerging as an important source of potable water, especially in areas with little rain but relatively high humidity.^1^ A good understanding of water condensation, a phase change process in which water vapor is transformed to liquid water and which involves both heat and mass transfers, is a key to an effective atmospheric water harvesting.

In a typical water condensation, there are three sequential steps: nucleation, growth of individual water droplet, and droplet coalescence.^2^ Nucleation is an initial step in forming water liquid from water vapor. There are two types of water nucleation: homogeneous and heterogeneous nucleation, with the former occurring in the absence of any foreign substrate while the latter occurring on foreign substrate with a temperature lower than vapor saturation temperature. Heterogeneous nucleation is a dominant mechanism in forming atmospheric water liquid under ambient conditions, and it is affected by parameters such as substrate temperature, vapor pressure, vapor temperature, and wettability of substrate. Generally, when the state of substrate and vapor is set, water vapor nucleates faster on a hydrophilic substrate than on a hydrophobic one due to its lower energy barrier.^3^ Following the nucleation is growth of an individual water droplet, and in this step, water vapor condenses on the preformed droplet surfaces and the mass of individual liquid droplet grows with time. When individual droplets grow larger, they tend to coalesce to minimize the total surface energy. Two important characteristics of the droplet coalescence stage are linear growth of droplet size on flat surfaces and constant water droplet surface coverage ratio, which is defined as ratio of the projected area of the droplets to the total substrate surface area.[[qv: 2a,4]] Both the growth of individual water droplet and droplet coalescence can be greatly influenced by aerodynamic parameters[[qv: 2a,5]] such as ambient air velocity.

Typically observed in our daily life is an edge effect that leads to faster water condensation and visually bigger water droplets on the substrate surface region near its edges than its central surface region.[[qv: 4b]] The edge effect is majorly due to special local aerodynamic conditions at the edges where higher air velocity can be expected, which facilitates growth rate of water droplets.[[qv: 2a,6]]

Once a coalesced droplet reaches a certain critical size, its gravity overcomes its retention force, the droplet would start to move downward along the surface, and the substrate surface is thus renewed for another water condensation cycle, a process known as substrate renewal or water droplet removal. Equation (1) determines the critical size of a water droplet that would initiate droplet downward movement on a flat substrate

(1) $$mg\;\times \;\sin\alpha \;=\;\gamma_{\mathrm{water}}\;\times \;(\cos\theta_{R}\;-\;\cos\theta_{A})\;\times \;W$$

where *mg* is the gravitational force from water mass, α is the tilting angle of substrate, γ_water_ is the water surface tension, θ_R_ and θ_A_ are water receding and advancing contact angles on the substrate, respectively, and *W* is the droplet width.^7^ Thus, a smaller contact angle hysteresis (CAH), which is defined as the difference between advancing and receding contact angles (i.e., θ_A_ − θ_R_), means a smaller critical droplet size. In order to have a high substrate renewal rate, a substrate with a low CAH is preferred, assuming other conditions being constant. A higher renewal rate in turn surely leads to a higher availability of substrate surface for water condensation and a quicker turnover of the surface. The above discussions are seemingly leading us to believe that a lower CAH would, in the end, result in a higher water mass collection rate, which is the ultimate goal of any practical atmospheric water harvesting. One major objective of this work is to prove that this seemingly reasonable argument is actually false.

In parallel with mass transfer, heat transfer is another important factor in water condensation as considerable amount of latent heat is released therein and the heat is transferred from the condensing water droplets to the substrate. Given the fact that water is a poor heat conductor with a heat conductivity of 0.6 W m^−1^ K^−1^ at 20 °C, heat transfer is generally the limiting factor in promoting water condensation and collection efficiency. It has been reported that dropwise condensation tends to have a higher heat transfer efficiency than filmwise condensation due to the heat barrier effect of water.^8^ Given the inherent complexity of heat transfer process, the research progress in heat transfer is lagging behind that of mass transfer in the general field of water condensation.^9^ However, recent and ongoing advancement in special wettability/morphology/composition surface preparation is providing scientists with unprecedented opportunities to better understand the heat transfer in the water condensation process.^9^, ^10^ Connecting mass transfer with heat transfer, it is expected that a fast water droplet removal surface guarantees a high heat transfer efficiency. For example, it has been reported that slippery surfaces with extremely low CAH enable fast removal of water droplet once condensed, which in turn leads to enhanced heat transfer efficiency.^11^

With a clear aim to enhance water mass collection efficiency, a great amount of research efforts have been made by scientists to investigate the water condensation and subsequent collection processes,^1^, ^5^ but there are certain fundamental aspects yet to be fully understood. First, fast water removal surface can be sometimes intuitively and wrongly taken for granted to lead to high water mass collection. Second, detailed studies on dynamics of water droplet growth and the kinetics of water mass collection are rare, which, if present, would provide valuable insights and thus considerably advance the current understanding of these processes. The aim of this work is, by carefully designed and prepared surfaces with different wettability and CAH, real‐time recorded droplet growth dynamics, continuously monitored water mass collection, and thoroughly conducted theoretical calculations, to provide convincing evidences to prove the insignificant role of surface wettability and more importantly CAH on water mass connection efficiency. The results of this work highlight the often overlooked but critical effect of the surface edge regions of a substrate on the growth of individual water droplets and thus overall water mass collection efficiency and thus show light on meaningful means of promoting atmospheric water harvesting efficiency for practical applications.

## 2 Results and Discussion

The present work focuses on water condensation on flat surfaces that are in contrast to surfaces with roughness. It is known that rough surfaces with proper surface chemistry assisted with micro/nanostructure geometry could exhibit unique and in many cases extreme wettability,^12^ but water condensation on such surfaces is always considerably more complicated than on their flat surface counterparts,[[qv: 10b,d,13]] with too many parameters coming to play. The scientific community is still at an early stage of its learning curve in fully grasping water condensation on rough surfaces. In contrast, water condensation on flat surfaces grows droplets in well‐known patterns referred to as “breath figure.”^14^ Thus, flat surfaces were rationally selected over rough ones in light of the goal of the project. We also believe that by choosing flat surfaces, the uncertainty in explaining the experimental results is considerably reduced and the manageability of the project is greatly raised. It is believed that the conclusions based on flat surfaces provide fundamental aspects to the same processes on rough surfaces and thus have trustworthy applicability to rough surfaces.

**Figure 1:**
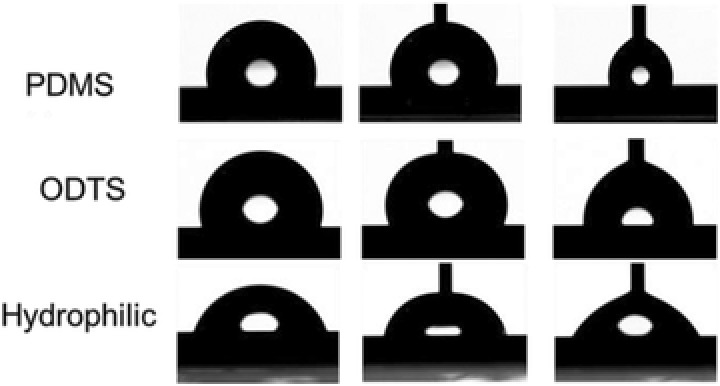
Digital images of the droplets on three surfaces during contact angle measurement (θ, θ_A_, and θ_R_ are on the left, in the middle, and on the right, respectively).

In the present work, three flat surfaces (namely, polydimethylsiloxane (PDMS)), octadecyltrichlorosilane (ODTS), and hydrophilic respectively) with contrasting contact angle and CAH were prepared. **Table** **1** presents the static contact angles, advancing contact angle, and receding contact angles of water on three different surfaces, while **Figure** **1** shows digital photos of water droplets during the contact angle measurements. As can be seen, the dimethyldimethoxysilane (DMS)‐modified surface presents a moderate hydrophilicity (θ = 72.0°) and a relatively high CAH (16.2°). The hydrophobic surfaces modified with PDMS and OTDS show similar static contact angles (101.6° and 107.0°, respectively), but CAH for the two surfaces differs from each other significantly (4.8° vs 19.8°). The low CAH of the PDMS‐modified surface arises from the liquid‐like property of the surface PDMS groups as the silicon–oxygen bond can easily rotate, while the high CAH of the ODTS‐modified surface is from the amorphous or crystal‐like property of the surface ODTS groups.^15^ Moreover, the atomic force microscopy (AFM) images show that roughness of all the surfaces prepared in this study is in sub‐nanometer range, indicating the flatness of the surfaces (Figure S1, Supporting Information).

**Table 1:** Summary of static and dynamic contact angle measurements, droplet growth rates, predicted droplet sliding diameter, and predicted and measured sliding time of all three surfaces

	Static contact angle [θ]	Advancing contact angle [θ_A_]	Receding contact angle [θ_R_]	CAH [°]	Predicted sliding diameter [mm]	Edge growth rate [μm s^−1^]	Nonedge area growth rate [μm s^−1^]	Predicted sliding time [s]	Measured sliding time [s]
PDMS	101.6 ± 1.0	103.0 ± 0.3	98.2 ± 0.4	4.8	1.34	0. 868	0.316	1544	1508 ± 251
Hydrophilic	72.0 ± 2.5	79.0 ± 0.9	62.8 ± 3.0	16.2	3.17	1.331	0.525	2382	2292 ± 429
ODTS	107.0 ± 1.1	113.3 ± 0.6	93.5 ± 1.4	19.8	2.34	0.719	0.270	3255	3314 ± 672

There exist a few reports on low CAH flat surfaces, one of which is so‐called SLIPS, which is inspired by the pitcher plant with lubricating oil infused into its surface nanostructures.^16^ There have been reports utilizing the SLIPS surfaces for water condensation and collection,^5^, ^11^, ^17^ but a disappointing result is that the water collected by such a surface was found to be a mixture of water and lubricating oil,[[qv: 1b]] which challenges the application stability of the SILPS. Water condensation experiments were conducted in a homemade, temperature‐ and humidity‐controlled chamber. In the chamber was a vertically placed, constant temperate cooling stage to which the modified silicon wafer substrate was attached. There is noteworthy characteristic of the substrate attachment onto the cooling stage: the left and right sides of the silicon wafer substrate were positioned within the cooling stage while the upper and lower sides being extended beyond the cooling stage. The special positioning of the silicon wafer substrate on the cooling stage helps reduce water condensation on the side cross‐sections of the left and right edge sides of the substrate, and the condensed water droplets therein are not collected on purpose by design. Moreover, the side cross‐sectional surface of the substrate's upper edge side is intentionally cut so that condensed water there would not fall off from the front face of the substrate (Figure S2, Supporting Information) and thus will not be counted and the water condensed on the bottom edge side's cross‐section would be drained and diverted away from the water collection vessel. This way, the edge effect from the bottom edge, left and right edge sides are all delicately eliminated so to fully focus our investigation on the upper edge of the substrate. All these purposeful designs are to make sure that only water condensed on the front surface of the modified silicon wafer are collected for the purpose of quantifying water collection efficiency by these special wettability surfaces. The growth dynamics of water droplets on the substrate surfaces are monitored by a microcamera.

**Figure 2:**
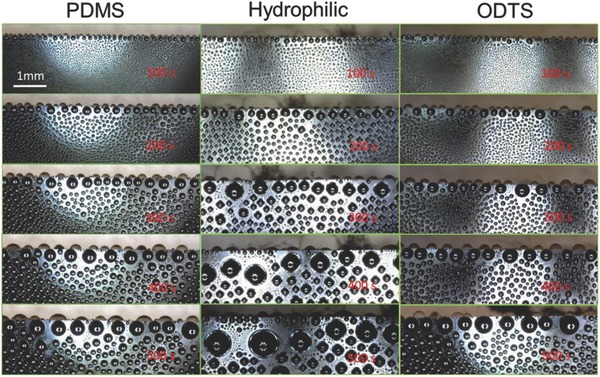
Images showing the growth of droplets on the edge and nonedge areas of the PDMS‐ and ODTS‐modified hydrophobic surfaces, along with the hydrophilic surface at the end of first 100, 200, 300, 400, and 500 s.

**Figure** **2** shows the images of water droplets growing on the different surfaces at the end of the first 100, 200, 300, 400, and 500 s. To facilitate discussion, the term “edge” thereafter refers to the linear edges of the front surface of modified silicon substrates. Some direct and major observations are as follows: (1) The droplets on the upper edges are always larger than those in the nonedge and central surface regions, regardless of surface wettability, which is a good proof of the edge effect on water condensation. (2) The droplets grow larger with time regardless of the locations on the surfaces (edge vs nonedge areas), the wettability (hydrophilic vs hydrophobic), and CAH of the surfaces. (3) The droplet size on the hydrophilic surface is always bigger than those on the hydrophobic surfaces (i.e., PDMS‐ and ODTS‐modified surfaces) at any time point. (4) For all of the surfaces, condensation follows dropwise condensation, indicated by well‐separated individual droplets on the surfaces at any time (100 to 500 s).

**Figure 3:**
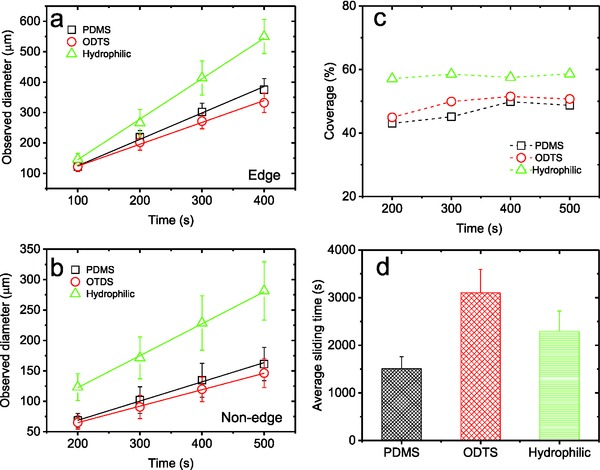
The observed droplet size as a function of time on a) the edge and b) the nonedge areas of the three surfaces; c) water droplet surface coverage ratios; d) measured average sliding time for the three surfaces. (Note: measurement of nonedge droplets started from 200 s because nonedge droplets in 100 s are too small).

In order to quantitatively describe the evolution of condensation on the substrates, the growth dynamics on the edges and in the nonedge areas are carefully examined. **Figure** **3**a,b presents the observed droplet sizes as a function of time on both the edge and nonedge areas, respectively, based on which the following three major observations are made. (1) It is clear that the droplet growth on all surfaces follows a linear relationship with time, which is in a good agreement with theory prediction and implies that the observation period (100–500 s) is within the droplet coalescence stage.[[qv: 2a,4b]] The linearly fitted growth rates are presented in Table 1. (2) It is also clear that the droplets grow much faster in size on the hydrophilic than on the PDMS‐ and ODTS‐modified hydrophobic surfaces on both edge and nonedge areas, while they grow similarly in size on the two hydrophobic surfaces, suggesting that hydrophilicity facilitates individual droplet growth. (3) More interestingly, the droplet growth rates on the edges for PDMS, ODTS, and hydrophilic are ≈2.7, 2.7, and 2.5 times faster than those on the nonedge areas of the same surfaces, which is a direct and very significant proof of the effect of edge on significantly facilitated water condensation and also indirectly points to the reliability and stability of our measurement system.

The water droplet surface coverage ratio (including edge and nonedge areas), which was defined previously, on the surfaces is then obtained based on the images from the recorded droplet growth dynamics following the literature method [[qv: 2a,4b]] and is presented as a function of time in Figure 3c.

The water coverage ratios remain relatively constant after a period of time (i.e., 200 s and beyond) and are ≈58%, 50%, and 50% for the hydrophilic, the PDMS‐, and POTS‐modified surfaces, respectively, indicating that water droplets cover larger area on a hydrophilic surface than on hydrophobic ones and that the water cover ratio is irrelevant to CAH.

As droplets continuously grow in size, there would be a droplet‐sliding process at one point. As the droplets on the edges grow much faster than those in the nonedge areas, the droplets always slide down from the edge to the bottom. The average sliding time for the hydrophilic, the PDMS‐, and ODTS‐modified surfaces is determined to be 2291, 1507, and 3314 s, respectively (Figure 3d).

After addressing two very important parameters in water condensation, droplet growth rate and droplet surface coverage ratio, we then turn to address some theoretical aspects of the process mathematically. Even though droplets condensing on the substrate are never uniform in size in reality, an assumed average size predicted from the droplet growth rate can still be used with reasonable accuracy in estimation when the variation in droplet size is not very wide, which is the case in this work (Figure 3a,b). Since the average droplet size could be predicted based on droplet growth rate, and droplet density (*N*, number of droplets per unit surface area) could be calculated by droplet surface coverage and droplet size, the total volume or mass of water condensed per unit surface area may thus be predicted based on the droplet growth rate and droplet surface coverage, both of which are known to us by now (Figure 3a–c).

**Figure 4:**
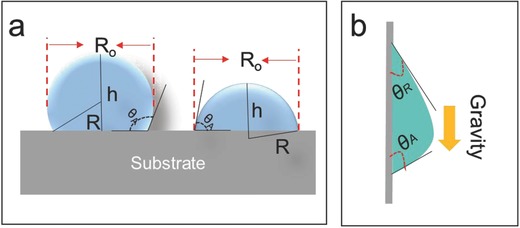
a) Schematic showing a single water droplet condensing on the hydrophobic (left) and hydrophilic (right) substrates. b) Schematic showing sliding of a water droplet on a vertically place substrate surface.

In more details, given the difference in surface wettability and thus in the geometry of the droplets on the surface, the hydrophobic and hydrophilic surfaces are treated differently. Assuming that a single water droplet is a partial sphere, the droplet sizes observed by the camera images for the hydrophobic surfaces are indeed the real droplet diameter (*R*_o_ of the left droplet in **Figure** **4**a), while the observed droplet sizes by the camera images for the hydrophilic surface are actually the width of the droplets (*R*_o_ of the right droplet in Figure 4a). Moreover, since the droplets are continuously growing in size during the condensation, advancing contact angle, along with the average droplet size (*R*_o_), is then used to calculate the volume of single droplet (*V*_single_) (Figure 4a). With *V*_single_ known, droplet density (*N*) could be calculated as the droplet surface coverage (Figure 3c) divided by the average droplet size (*R*_o_). At last, the total water volume per unit substrate surface area ($\bar{V}$) is calculated by Equation (2)

(2) $$\bar{V}\;=\;V_{\mathrm{single}}\;\times \;N$$

The detailed mathematical calculations can be found in the Supporting Information.

Interestingly, the calculated growth rate of condensed water volume $\left(\frac{d\bar{V}}{dt}\right)$ per unit area of both the edge and nonedge areas shows relatively independence on the surface wettability and CAH of these surfaces. However, $\frac{d\bar{V}}{dt}$ for the edges of PDMS‐, ODTS‐modified, and the hydrophilic surfaces is constantly 2.7, 2.7, and 2.5 times than that for the nonedge areas of the same surfaces, consistent with the water droplet growth rates as previously reported (Figure 3a,b). This discovery worrisomely implies that water collection rate might be the same for surfaces with different contact angles and thus wettability.

As presented previously, hysteresis affects droplet‐sliding time on these surfaces. The critical size (*D*) of a droplet, beyond which the droplet moves on a vertically placed surface under an influence by gravity (as shown in Figure 4b), is theoretically determined by Equation (3)

(3) $$D=2\;\times \;\sqrt{\frac{6\gamma_{\mathrm{water}}\times \;\sin\theta_{A}\times \;(\cos\theta_{R}-\;\cos\theta_{A})}{\pi \;\times \;\rho \;\times \;g\;\times \;{\left(1\;-\;\cos\theta_{A}\right)}^{2}\;\times \;(2\;+\;\cos\theta_{A})}}$$

where ρ is the density of droplet and *g* is the gravity constant.

The predicted critical droplet sizes, determined from the camera images, are 3.17, 1.34, and 2.34 mm for the hydrophilic, PDMS‐, and OTDS‐modified hydrophobic surfaces, respectively (Table 1), indicating that smaller CAH allows for smaller water droplets to move on surface. As the water droplets grow faster and bigger on the edges than on the nonedge areas and the critical droplet size for sliding (*D*) is uniformly the same across the entire surface irrespective of edge and nonedge areas, it is thus always the droplets grown on the edge area that slide down first from the top edge of the substrate surface and sweep away the droplets present in their paths of downward movement on the surface. Based on this, the sliding time, the time point when the droplets initiate their movement, of the droplets at the edge area is then calculated to be 2260, 1540, and 2840 s for the hydrophilic, PDMS‐, and ODTS‐modified surfaces, respectively. The calculated sliding time matches well with the measured ones for all three surfaces, which again indirectly confirms the reliability and stability of our condensation system. The results seem to suggest that the droplets grow only by the rate measured at the first 500 s and the high hysteresis does not slow down the droplet growth rate in a later stage. In other words, our results conclude that the droplet growth rates are independent of the surface CAH in this work.

However, this does not lead to a general conclusion that hysteresis would not affect droplet growth rate at all. As a matter of fact, with a considerably high hysteresis of a surface, we are uncertain whether a different scenario would be happening given the possibility that heat transfer may play a bigger role there as the high hysteresis would facilitate heat transfer to substrate. Nevertheless, our results clearly indicate that the heat transfer factor, which is related to surface CAH, is not a significant player in water condensation under the conditions employed in this work.

**Figure 5:**
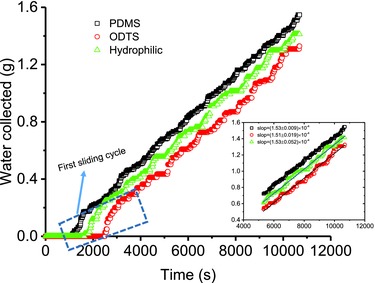
Condensation water mass collection kinetics of the PDMS‐ and ODTS‐modified hydrophobic surfaces and the hydrophilic one. Note: the box on the figure delineates the initial unstable zone, while the inset figure shows the kinetics of the stable stages on the three surfaces.

**Figure** **5** presents the time course of the measured water mass collected. There is an initial period where the rate is not stable while the first sliding cycle is taking place, and as time goes on, stable rates are achieved on all surfaces. Interestingly and somewhat as expected, after an initial stabilization period, water mass collected increases linearly with time with the slopes for all three surfaces being statistically the same (the inset in Figure 5). Thus, our result sadly indicates that surfaces with different surface wettability and hysteresis tend to have the same water mass collection rate, which agrees well with the theoretical calculations in this work. However, it is worth pointing out that, for a surface with a lower hysteresis, it allows for a smaller droplet to slide down and thus leads to a faster surface turnover, and the water mass collected at any time point is always greater than that on a surface with a higher hysteresis.

## 3 Conclusion

In conclusion, surfaces with contrasting CA and CAH were prepared and the condensation experiments were conducted on these surfaces. It turned out that water droplets on the edges always grew faster than those in the nonedge area. However, both the theoretical calculations and experimental observations conducted in this work showed that the total water mass collection rate by the different surfaces was comparable, independent of surface wettability and hysteresis. During water condensation, a balance has to be struck between single droplet growth and droplet density on a surface so as to maintain a constant water droplet surface coverage ratio, which, we believe, renders the role of both wettability and hysteresis insignificant. The results of this work unequivocally point out the importance of increasing fraction of edge or pseudo‐edge structures which would promote locally favorable aerodynamics and rough structures that support removal of condensed droplets to increase effective condensation area on water condensation surfaces. For example, surfaces mimicking the cactus would be highly desired.

## 4 Experimental Section

*Materials*: DMS (95%), sulfuric acid (95–98%), and ODTS (98%) were purchased from Sigma‐Aldrich. 2‐Isopropanol (electrochemical grade) was purchased from Fisher Scientific. Ethanol absolute was purchased from VWR International. Deionized (DI) water was used in all of the experiments.

*Preparation of Surfaces with Different Contact Angles and CAH*: Polished silicon wafer of 54 mm × 50 mm × 0.625 mm was degreased with ethanol by sonication and then treated by oxygen plasma. Hydrophobic surface with low CAH, denoted as PDMS‐modified surface, was prepared on the polished front side of the plasma‐cleaned silicon wafer following a literature method.^18^ Briefly, an aliquot of 2.85 mL DMS, 31.80 mL isopropanol, and 135 μL sulfuric acid were mixed together gently, and the mixture solution was let to sit still for 30 min before use. The silicon wafer was then immersed in the mixture solution for 10 s before being taken out for drying in ambient air with a relative humidity of 60% and a constant temperature at 21 °C for 30 min. After drying, the substrate was washed with copious ethanol and DI water.

Hydrophobic surface with high CAH, denoted as ODTS‐modified surface, was prepared by immersing the plasma‐cleaned silicon wafer in 5 × 10^−3^ m ODTS toluene solution for 10 min, followed by washing with ethanol and water repeatedly.

Moderately hydrophilic surface, denoted as hydrophilic surface, was prepared by chemical vapor deposition of DMS on the precleaned silicon wafer under 70 °C for 1.5 h in a 100 mL container, followed by heating at 80 °C for 1 h to evaporate loosely bound DMS.

*Characterization of Surfaces*: Static contact angles were measured with a commercial contact angle system (OCA 35, DataPhysics, Filderstadt, Germany) at ambient temperature using a 4 μL water droplet as a probe. Advancing and receding contact angle measurements were conducted by adding and withdrawing the probe water with a speed of 0.5 μL s^−1^, respectively. Each contact angle value reported was an average of four individual measurements at different locations on the same surfaces. AFM images were taken by Agilent 5500 SPM using tapping mode.

**Figure 6:**
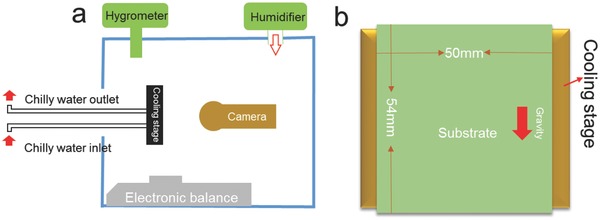
Schematic of a) homemade water condensation and collection experimental setup, and b) special positioning of the modified silicon wafer substrates attached on the cooling stage.

*Water Condensation Experiments*: **Figure** **6**a is a schematic showing the condensation experimental setup (images also shown in Figure S3a in the Supporting Information). Briefly, condensation experiment was conducted in a homemade humidity chamber with a stable environmental temperature at 21 °C. The relative humidity inside the chamber was maintained at 100% by continuous moisture supply from a commercial ultrasonic humidifier.

In the humidity chamber was a vertically placed cooling stage to which the modified silicon wafer substrate was attached. The cooling stage was maintained at a constant temperature at 4 °C by circulating chilly water. As presented in Figure 6b and Figure S3b (Supporting Information), the left and right sides of the substrate were kept within the cooling stage while the upper and down sides being extended out of the cooling stage.

Growth dynamics of water droplets on the substrate surfaces was real time recorded by a microcamera (Dinocapture 2.0) connected with a PC, and the mass of water collected by the substrate surfaces was monitored by an electronic balance connected with a PC. The water droplets pinned at the bottom of the substrate surfaces were drained away from the condensed water collection container by a copper mesh covered with cotton that was placed very close to the bottom of the substrate surface but falling short of touching it.

## Conflict of Interest

The authors declare no conflict of interest.

## Supporting information

Supplementary
