# Supplementary material for: Atmospheric Water Harvesting: Role of Surface Wettability and Edge Effect
Source: Glob Chall. 2017 Jun 23;1(4):1700019. doi: 10.1002/gch2.201700019 (PMC6607251; doi:10.1002/gch2.201700019)
Supplement: Supplementary file 1 — Supplementary [file GCH2-1-1700019-s001.pdf]

Copyright WILEY-VCH Verlag GmbH & Co. KGaA, 69469 Weinheim, Germany, 2017.

Supporting Information

for *Global Challenges,* DOI: 10.1002/gch2.201700019

Atmospheric Water Harvesting: Role of Surface Wettability and Edge Effect

Yong Jin, Lianbin Zhang, and Peng Wang**

Supporting Information

Atmospheric Water Harvesting: Role of Surface Wettability and Edge Effect

Yong Jin, Lianbin Zhang, Peng Wang**

1 *Water Desalination and Reuse Center, Division of Biological and Environmental Science and Engineering, King Abdullah University of Science and Technology, Thuwal 23955-6900, Saudi Arabia*

2 *Key Laboratory of Materials Chemistry for Energy Conversion and Storage (HUST) of Ministry of Education, School of Chemistry and Chemical Engineering, Huazhong University of Science and Technology, Wuhan 430074, China*

*Corresponding authors: zhanglianbin@hust.edu.cn and peng.wang@kaust.edu.sa

S1 AFM images of PDMS, ODTS and hydrophilic surfaces.

AFM images were taken to show surface roughness of the three kinds of surfaces. The root mean squared roughness (R_q) for PDMS, ODTS and hydrophilic surfaces are 0.85, 0.88 and 0.73 nm, respectively. The roughness parameters verified the flatness of the as-prepared surfaces.

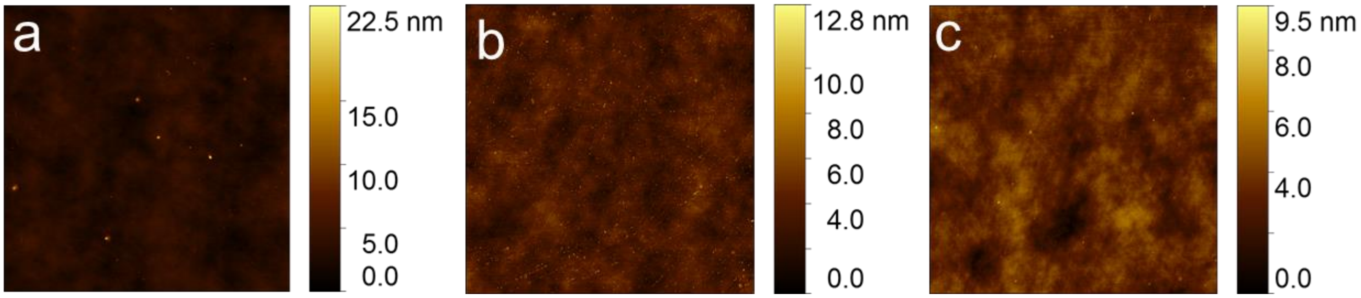

Figure S1. AFM images of (a) PDMS, (b) ODTS and (c) hydrophilic surfaces. The dimension of images is 10μm×10 μm.

S2 Schematic showing the design of upper side cross section of silicon wafer.

The upper side cross section of the silicon wafer was intentionally cut so that water droplets condensed on the cross section would fall off toward the back side. This design ensures that water collection on the front side would not be affected by the upper side cross section.

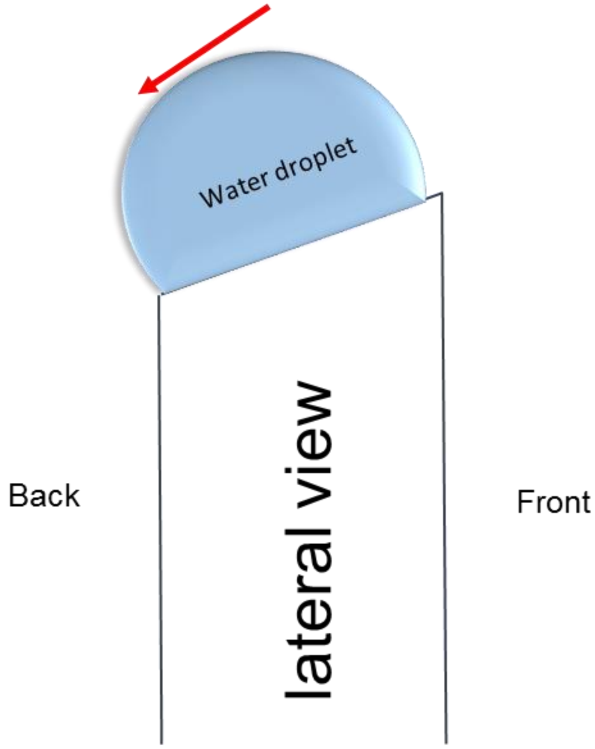

Figure S2. Schematic showing the design of upper side cross section of silicon wafer.

S3 Experimental setup and position of substrate on the cooling stage.

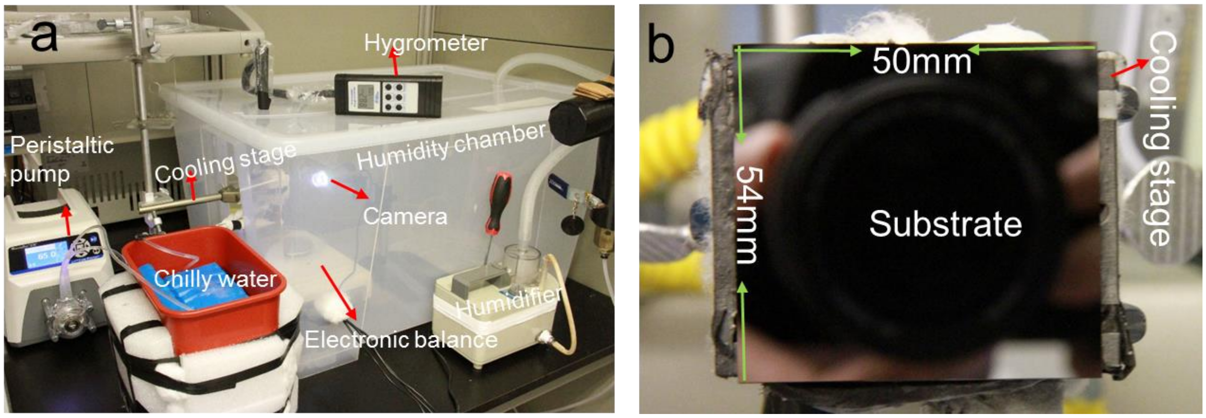

Figure S3. (a) Images of condensation experimental setup; (b) Images of substrate attached to the cooling stage.

S4 Calculation of volume of single water droplet and volume of total condensed water on a substrate surface

The volume of a single water droplet can be calculated as:

$$V_{single} = \pi h^2 \times \left(R - \frac{h}{3}\right)$$

$$h = R\ (1 - \cos\theta_A)$$

For hydrophobic

$$V_{hydrophobic} = \frac{\pi {R_o}^3 (1 - \cos\theta_A)^2 \times (2 + \cos\theta_A)}{3}$$

For hydrophilic

$$V_{hydrophilic} = \frac{\pi {R_o}^3 \times (1 - \cos\theta_A)^2 \times (2 + \cos\theta_A)}{3\sin{\theta_A}^3}$$

Droplet density can be calculated as:

$$N = \frac{\varepsilon^2}{\pi \times {R_o}^2}$$

Total volume of condensed water per unit are can be calculated as:

$$\bar{V} = V_{single} \times \mathrm{N} = \frac{R_o \times \varepsilon^2 \times (1 - \cos\theta_A)^2 \times (2 + \cos\theta_A)}{3}$$

$$\text{or} = \frac{R_o \times \varepsilon^2 \times (1 - \cos\theta_A)^2 \times (2 + \cos\theta_A)}{3\sin{\theta_A}^3}$$

When surface coverage is stable,

$$\frac{d\bar{V}}{dt} = Constant \times \frac{dR_o}{dt},$$

where *constant*=$\varepsilon^2 \times (1 - \cos\theta_A)^2 \times (2 + \cos\theta_A)/3$ for hydrophobic surface

or *constant*=$\frac{\varepsilon^2 \times (1-\cos\theta_A)^2 \times (2+\cos\theta_A)}{3 \sin\theta_A{}^3}$ for hydrophilic surface.

If we apply the measured parameters including S,θ_A, and growth rate in the non-edge areas, we can have:

$\frac{d\overline{V_{PDMS}}}{dt}$=0.14

$\frac{d\overline{V_{ODTS}}}{dt}$=0.14

$\frac{d\overline{V_{Hydrophilic}}}{dt}$=0.15.

The growth rate of droplets on the edges of PDMS, ODTS and Hydrophilic was around 2.7, 2.7 and 2.5 times of those on the non-edge area.

Summary of volume growth rate on edge and non-edge

$\frac{d\bar{V}}{dt}$	Edge	Non-edge
PDMS	0.38	0.14
ODTS	0.38	0.14
Hydrophilic	0.38	0.15
